# Polygenic Collagen Variants and Their Association With Ligament Injury Patterns in Patients With Knee Ligament Injuries

**DOI:** 10.7759/cureus.103472

**Published:** 2026-02-12

**Authors:** Jurgen Frese-Arroyo, Santiago De la Garza-Castro, José F Vílchez-Cavazos, Victor M Peña-Martínez, Jorge Lara-Arias

**Affiliations:** 1 Orthopedics and Traumatology Service, “Dr. José Eleuterio González” University Hospital, Universidad Autónoma de Nuevo León, Monterrey, MEX

**Keywords:** anterior cruciate ligament, collagen polymorphisms, knee ligament injury, polygenic susceptibility, sports-related knee injury

## Abstract

Knee ligament injuries, particularly anterior cruciate ligament (ACL) tears, represent a major cause of functional limitation in physically active individuals. While biomechanical and environmental factors are well-established contributors, increasing evidence suggests that genetic variability in collagen-related genes may influence individual susceptibility to ligament injury. Most previous studies, however, have focused on elite athletic populations, limiting their applicability to routine clinical settings. A prospective cross-sectional study was conducted in 94 adult patients with clinically and radiologically confirmed knee ligament injuries. The study was conducted at the University Hospital ‘Dr. José Eleuterio González’ (Hospital Universitario “Dr. José Eleuterio González”), Universidad Autónoma de Nuevo León (UANL), Monterrey, Mexico, a tertiary care university hospital. All participants completed a structured clinical questionnaire addressing demographic characteristics, sports participation, and injury mechanism. Genomic DNA was obtained from buccal swabs, and genotyping of the rs1107946 (COL1A1), rs42524 (COL1A2), and rs1800255 (COL3A1) polymorphisms was performed using real-time polymerase chain reaction. In addition to individual variant analysis, a polygenic score was calculated by integrating the presence of risk-associated genotypes. The study population had a mean age of 28.4 years, with a predominance of male patients and sports-related injuries. Most injuries occurred through non-contact mechanisms and involved complete ACL rupture. Individually, none of the analyzed polymorphisms showed a significant association with injury severity. However, patients with multiligament injuries and those with non-contact injury mechanisms exhibited a significantly higher polygenic burden compared with patients presenting isolated ACL injuries or contact-related mechanisms. These findings suggest that susceptibility to knee ligament injuries is influenced by a polygenic genetic component rather than isolated variants, supporting a multifactorial approach to injury risk assessment and highlighting the potential role of genetic factors in personalized prevention strategies.

## Introduction

Knee ligament injuries are among the most frequent musculoskeletal conditions in physically active populations and represent a substantial source of morbidity, functional limitation, and healthcare utilization. Anterior cruciate ligament (ACL) rupture is strongly associated with sports participation and typically affects young, active individuals, often requiring prolonged rehabilitation and, in many cases, resulting in persistent joint instability or early degenerative joint changes [1-3].

Epidemiological evidence has consistently shown that a considerable proportion of ACL injuries occur without direct contact, most often during rapid deceleration, pivoting, or landing maneuvers [4,5]. Although biomechanical factors such as neuromuscular control, lower-limb alignment, and training load play a central role, these mechanisms alone do not fully explain why individuals exposed to comparable athletic demands experience markedly different injury outcomes.

From a tissue perspective, ligaments are composed predominantly of type I and type III collagen, which together confer tensile strength, elasticity, and resistance to mechanical stress [6]. Disruptions in collagen synthesis, fibrillar organization, or the relative proportion of these collagen types may compromise ligament integrity and increase the likelihood of failure under physiological loads [6,7]. In this context, genetic polymorphisms in collagen-encoding genes have been proposed as contributors to interindividual variability in ligament structure and injury susceptibility [8-11].

Prior studies have reported associations between variants in collagen type I alpha 1 chain (COL1A1), collagen type I alpha 2 chain (COL1A2), and collagen type III alpha 1 chain (COL3A1) and ligament or tendon injuries, particularly in athletic cohorts [8-11]. Variants within COL3A1 have been linked to changes in collagen elasticity, whereas polymorphisms in COL1A1 and COL1A2 may influence fibrillar organization and tensile properties [8-11]. However, findings across studies have been heterogeneous, and many investigations have assessed single polymorphisms in isolation, which may underestimate the cumulative genetic contribution to ligament vulnerability [8-11].

Increasingly, research in musculoskeletal genetics supports a polygenic model in which multiple low-impact variants collectively contribute to tissue resilience and injury susceptibility [12,13]. This framework may be especially relevant for ligament injuries, where structural failure often reflects a complex interaction of biological, mechanical, and environmental factors rather than a single dominant cause.

Accordingly, the present study aimed to evaluate the relationship between polymorphisms in COL1A1, COL1A2, and COL3A1 and the clinical presentation of knee ligament injuries in a heterogeneous clinical population. In addition, we explored whether a cumulative polygenic burden is associated with injury complexity and mechanism, with the goal of generating clinically meaningful insights applicable to routine orthopedic practice.

## Materials and methods

Ethical approval

The study was conducted at the University Hospital ‘Dr. José Eleuterio González’ (Hospital Universitario “Dr. José Eleuterio González”), Universidad Autónoma de Nuevo León (UANL), Monterrey, Mexico, a tertiary care university hospital. This study was reviewed and approved by the Research Ethics Committee of the institution (approval number: OR24-00011). All procedures were conducted in accordance with institutional and national ethical standards and the Declaration of Helsinki.

Study design and population

A prospective, cross-sectional study was conducted in adults with knee ligament injuries evaluated at a tertiary care university hospital, reflecting routine orthopedic and trauma practice in a clinically heterogeneous population. Ninety-four patients were enrolled after meeting eligibility criteria and providing written informed consent. All participants had a clinically and/or imaging-confirmed ligament injury involving the ACL, posterior cruciate ligament (PCL), medial collateral ligament (MCL), lateral collateral ligament (LCL), or combinations of these structures.

Eligibility criteria

Patients aged ≥18 years of either sex with a confirmed knee ligament injury were eligible. Exclusion criteria were high-energy trauma (e.g., motor vehicle accidents or falls from height), known hereditary connective tissue disorders, prior knee reconstructive surgery unrelated to the index injury, significant angular deformity, or any condition that could compromise reliable data collection or buccal DNA sampling.

Clinical data collection

Clinical and demographic data were obtained using a structured questionnaire administered after written informed consent (see Appendix). The questionnaire was developed by the authors (orthopedic clinicians) for this study, based on routine clinical assessment and commonly reported variables in knee ligament injury practice; it was not adapted from a previously published instrument. Items captured age, sex, level, and type of physical activity, injury mechanism (contact vs non-contact), sport or activity at the time of injury, ligament(s) involved, injury severity, treatment received, and history of prior ligament injuries. These data were used to describe the cohort and to explore associations between clinical variables and genetic findings.

DNA sampling and extraction

Genomic DNA was collected using buccal swabs from the inner cheek. Samples were processed with a semi-automated magnetic bead-based extraction system (Zybio® EXM3000, Zybio Inc., Chongqing, China) according to the manufacturer’s instructions. DNA concentration and purity were assessed spectrophotometrically, and extracts were stored at −20 °C until analysis.

Genotyping by real-time PCR

Genotyping of rs1107946 (COL1A1), rs42524 (COL1A2), and rs1800255 (COL3A1) was performed using real-time polymerase chain reaction (PCR) with allele-specific primers and probes, following previously validated protocols [12,14,15]. Amplification was carried out on a QuantStudio™ 5 Real-Time PCR System (Thermo Fisher Scientific, USA) using a commercial genotyping master mix. Primer sequences were as follows: COL1A1 (rs1107946), forward 5′-CCTACTGTGGGTCAGTTCCAAGAGA-3′ and reverse 5′-CCCCTCCCTAATAGGCGACAGGGT-3′; COL1A2 (rs42524), forward 5′-AGGTGGAAAAGGTGAACAGGGTCCC-3′ and reverse 5′-CTGGTCCTCCAGGCTTCCAGGTAAG-3′; and COL3A1 (rs1800255), forward 5′-TGGTGAACGTGGACCTCCTGGATTG-3′ and reverse 5′-GGTGAATGGAATGCTGTGGAGTTACCTTT-3′. Reactions were performed in a final volume of 20 µL containing master mix, forward and reverse primers, allele-specific probe, approximately 20 ng of genomic DNA per reaction, and nuclease-free water. Cycling conditions were 95 °C for 10 minutes, followed by 40 cycles of 95 °C for 15 seconds and 60 °C for 60 seconds. All samples were analyzed in duplicate, and positive and negative controls were included in each run. Allelic discrimination was performed using the instrument software, and results were exported for statistical analysis.

Polygenic risk assessment

Genotypes were categorized as homozygous wild-type, heterozygous, or homozygous variant. A polygenic risk score was calculated by assigning numerical values according to the number of risk alleles per polymorphism and summing across COL1A1, COL1A2, and COL3A1, allowing assessment of combined genetic burden rather than isolated variants. Higher scores reflected greater cumulative genetic risk.

Statistical analysis

Analyses were performed using IBM SPSS Statistics version 25.0 (released 017, IBM Corp., Armonk, NY, USA). Continuous variables are presented as mean ± standard deviation or median (interquartile range), as appropriate. Categorical variables are reported as frequencies and percentages. Comparative analyses assessed associations between genetic findings (including polygenic score) and clinical injury characteristics. A two-sided p-value < 0.05 was considered statistically significant.

## Results

Study population characteristics

A total of 94 patients with confirmed knee ligament injuries were included in the analysis. The mean age was 28.4 years, with a predominance of male participants. Most injuries occurred during physical or sports-related activities, reflecting an active cohort. Non-contact mechanisms were more frequent than contact-related injuries (Table 1).

**Table 1:** Demographic and clinical characteristics of the study population Values are presented as mean ± standard deviation (range) or n (%). ACL: anterior cruciate ligament.

Variable	Category	Result
Total participants	Overall	94
Age (years)	Mean ± SD	28.4 ± 11.1
	Range	18-68
Sex	Male	64 (68.1%)
	Female	30 (31.9%)
Activity at the time of injury	Sports	78 (82.9%)
	Fall	9 (9.6%)
	Accident	7 (7.4%)
Injury mechanism	Non-contact	81 (86.2%)
	Contact	13 (13.8%)
ACL rupture	Present	94 (100%)
ACL tear grade	Complete	65 (69.3%)
	Partial	29 (30.7%)
Injury pattern	Isolated ACL injury	77 (81.9%)
	Multiligament injury	17 (18.1%)

Overall, the cohort profile aligns with prior reports in physically active populations and is compatible with genotype distributions typically observed in large population-based genomic reference datasets [16].

Distribution of genetic variants

Genotyping showed a predominance of heterozygous genotypes across the three evaluated loci (COL1A1, COL1A2, and COL3A1), including variants previously described as risk-associated. The most frequent genotypes corresponded to reference alleles commonly reported in the general population, whereas lower-frequency genotypes included heterozygous and homozygous variant profiles.

When each polymorphism was assessed individually, no single variant showed a clear independent association with injury severity or ligament involvement. Nevertheless, the observed genotype distribution supported subsequent analyses based on a combined polygenic approach (Table 2).

**Table 2:** Distribution of collagen gene polymorphisms in the study population Data are presented as mean ± standard deviation. COL1A1: collagen type I alpha 1 chain; COL1A2: collagen type I alpha 2 chain; COL3A1: collagen type III alpha 1 chain; ACL: anterior cruciate ligament; n: number of participants; SD: standard deviation; p: p-value. Statistical comparison performed using Student’s t-test. A p-value < 0.05 was considered statistically significant.

Gene (polymorphism)	Genotype	n (%)	Interpretation
COL1 A1 (G>T)	GG	35 (37.2%)	Most common genotype
	GT	44 (46.8%)	Heterozygous; intermediate genotype / risk trend reported
	TT	15 (16.0%)	Less frequent; increased risk described in athletic cohorts
COL1 A2 (G>C)	GG	46 (48.9%)	Most common genotype
	GC	34 (36.2%)	Heterozygous; intermediate genotype / associated with overload-related injuries
	CC	14 (14.9%)	Least frequent; associated with connective tissue fragility and multiligament injuries
COL3 A1 (G>A)	GG	30 (31.9%)	Most common genotype
	GA	31 (33.0%)	Heterozygous; intermediate genotype
	AA	33 (35.1%)	Risk genotype described in sports-related ligament injuries

Polygenic risk distribution

The cumulative polygenic risk score, defined as the total number of risk alleles across COL1A1, COL1A2, and COL3A1, showed that most participants clustered within intermediate values, most commonly between two and four risk alleles. This pattern reflects adequate genetic variability within the cohort and avoids overrepresentation of extremely low- or high-burden profiles.

This balanced distribution enabled meaningful comparisons across clinical subgroups and injury characteristics, supporting subsequent analyses examining the relationship between genetic burden, injury type, and injury mechanism.

**Figure 1:**
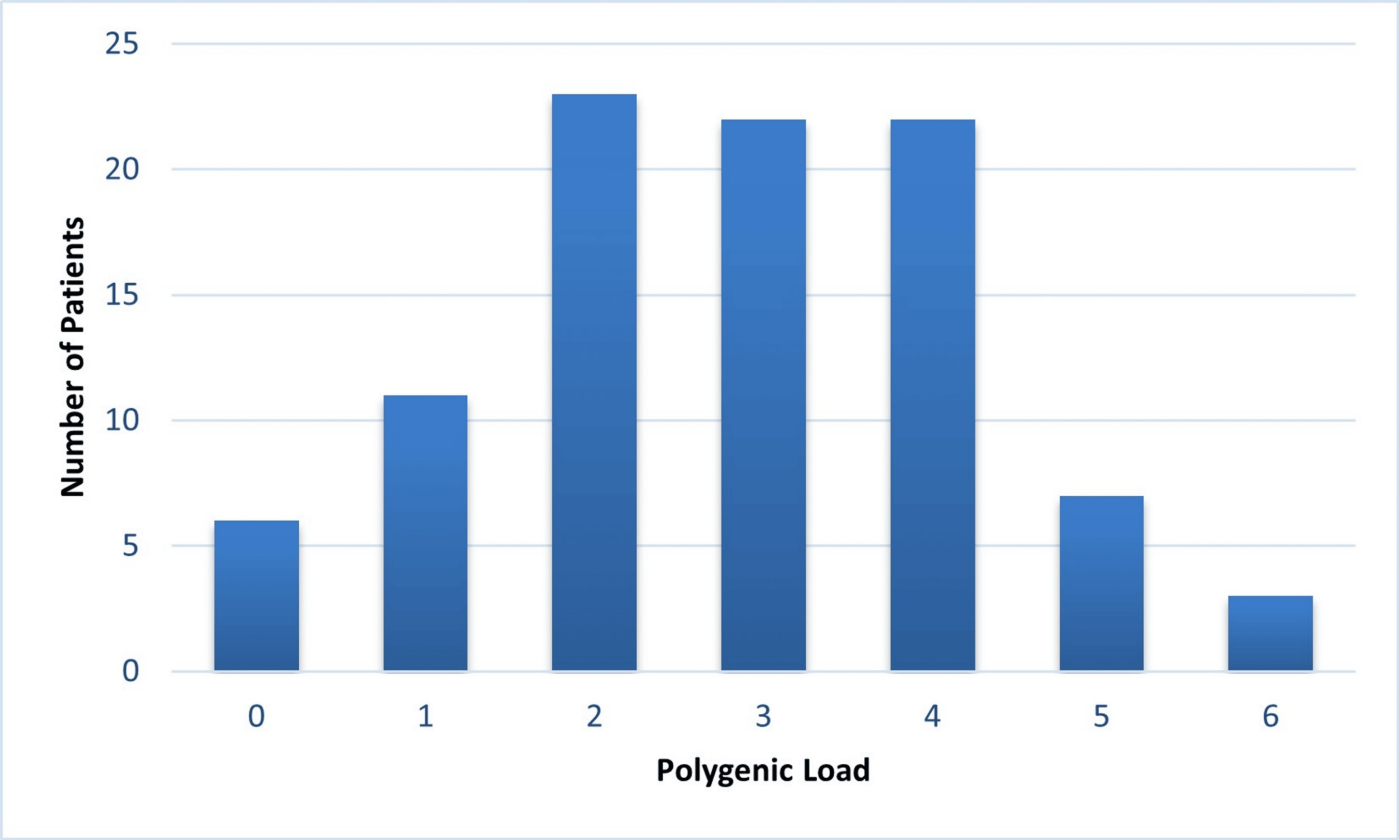
Distribution of polygenic load in the study population. The histogram shows the number of patients according to the cumulative number of risk alleles identified across the COL1A1, COL1A2, and COL3A1 genes. Most participants clustered within intermediate polygenic load values (2–4 risk alleles), indicating adequate genetic variability within the cohort.

Figure 1 illustrates the distribution of the cumulative polygenic risk score in the study population, expressed as the total number of risk alleles identified across the three collagen-related genes.

Polygenic risk and type of ligament injury

When polygenic risk was compared by injury pattern, patients with multiligament involvement exhibited a higher cumulative number of risk alleles than those with isolated ACL injuries. Statistical testing demonstrated a significant difference between groups.

This finding suggests that the accumulation of collagen-related risk variants may be associated with greater susceptibility to complex ligament injury patterns, rather than isolated ACL disruption. In practical terms, individuals with injuries affecting more than one ligament tended to show a higher genetic burden involving collagen type I and III, which may translate into reduced structural stability of ligament tissue under functional loading.

Table 3 summarizes the comparison of cumulative polygenic risk scores between patients with isolated ACL injuries and those with multiligament injuries (analysis performed on participants with available polygenic-score data).

**Table 3:** Comparison of cumulative polygenic risk according to type of ligament injury. Patients with multiligamentary injuries demonstrated a significantly higher cumulative polygenic risk score compared with those presenting isolated ACL injuries.

Type of injury	n	Polygenic risk score (mean ± SD)	p
Isolated ACL injury	77 (81.9%)	2.61 ± 1.39	0.0066
Multiligamentary injury	17 (18.1%)	3.65 ± 1.41	

Polygenic risk and mechanism of injury

Comparison by injury mechanism showed a clear pattern: patients who sustained ligament injuries through non-contact mechanisms had, on average, higher cumulative polygenic risk scores than those whose injuries occurred through direct contact.

This pattern supports the concept that collagen-related genetic variability may contribute to ligament failure under routine biomechanical demands, particularly during sports movements that involve deceleration, pivoting, or abrupt changes in direction. By contrast, contact-related injuries appeared to be less dependent on baseline tissue susceptibility and more strongly driven by external traumatic forces.

To quantify this association, cumulative polygenic risk scores were compared between contact and non-contact injury mechanisms. As shown in Table 4, the non-contact group demonstrated a significantly higher polygenic burden, reinforcing the hypothesis that genetic predisposition may be more influential in low-energy injury scenarios.

**Table 4:** Comparison of cumulative polygenic risk according to injury mechanism. Patients with non-contact knee ligament injuries exhibited a significantly higher cumulative polygenic risk score compared with those sustaining contact-related injuries.

Injury mechanism	n	Polygenic risk score (mean ± SD)	p
Contact	13 (13.8%)	1.83 ± 1.19	0.00023
Non-contact	81 (86.2%	2.97 ± 1.33	

## Discussion

The present study evaluated the potential association between polymorphisms in extracellular matrix-related genes (COL1A1, COL1A2, and COL3A1) and knee ligament injuries in adult patients with a history of recreational or sports-related physical activity. In contrast to prior investigations focused mainly on elite or professional athletes, a key strength of this work is the inclusion of a heterogeneous cohort that more closely reflects routine orthopedic and trauma practice. This strengthens the external validity of the findings for everyday clinical settings beyond highly selected athletic cohorts.

From a biological standpoint, evidence from large-scale population genomics suggests that the clinical relevance of many variants lies less in isolated effects and more in their cumulative contribution to tissue integrity and functional resilience. Analyses of mutational constraint across diverse human populations support the concept that multiple low-impact variants may collectively influence susceptibility to complex phenotypes, particularly in structurally dependent tissues such as connective tissue [17].

In our cohort, most injuries occurred during recreational sports and were associated with non-contact mechanisms. Sudden deceleration, pivoting, and landing maneuvers have been repeatedly identified as predominant injury mechanisms, even in the absence of direct trauma [18,19]. This observation supports the concept that, beyond mechanical factors, intrinsic biological determinants may influence how ligament tissue responds to functional loading under everyday athletic demands.

Regarding genetic findings, the distribution of COL3A1 rs1800255 (G>A) genotypes was comparable to that reported in international literature. Stepien-Słodkowska et al. described an overrepresentation of the AA genotype among Polish skiers with rupture, suggesting a relevant role of type III collagen in ligament elasticity and resistance [20]. Similarly, Lince et al. reported a higher frequency of risk-associated variants in South African athletes with ligament injuries, reinforcing the notion that alterations in collagen composition may predispose individuals to injury under repetitive or submaximal loading conditions [14].

Unlike studies restricted to highly selected athletic cohorts, the present investigation included individuals with varying levels of physical activity, allowing assessment of the clinical expression of these polymorphisms in a more generalizable context. The findings suggest that collagen-related genetic variability may contribute to ligament injury susceptibility beyond elite sport settings and may also be relevant in recreationally active individuals.

For COL1A1 and COL1A2, our results were consistent with prior work reporting modest but reproducible associations between type I collagen variants and injury risk. Studies in European and South American athletic populations have suggested that COL1A1 (rs1107946) and COL1A2 (rs42524) variants may alter fibrillar organization and tensile properties, potentially increasing susceptibility to injury from repetitive stress or overload [12,21]. In the present study, although individual polymorphisms did not show strong independent associations, combined analysis suggested a tendency toward higher genetic burden in patients with more complex or combined ligament injuries.

One of the most relevant findings was the utility of a composite polygenic risk variable integrating multiple collagen-related polymorphisms. This approach aligns with contemporary genomic research, indicating that susceptibility is unlikely to depend on a single variant and may instead reflect the cumulative interaction of multiple loci involved in connective tissue structure and remodeling [22]. In this context, the multigene association analysis by Kim et al. supports models that consider the aggregated contribution of several variants to ligament injury susceptibility [23].

Clinically, these findings are not intended to support immediate diagnostic or screening applications of genetic testing. Rather, they provide exploratory evidence that may help explain interindividual variability in ligament response under comparable mechanical loads. Prior studies on risk factors for re-injury have emphasized variables such as age, graft type, and activity level, while acknowledging an unexplained component that may have a biological basis [24,25]. In the future, integrating genetic information could contribute to more refined risk stratification models and individualized preventive strategies.

Several limitations should be acknowledged, including sample size and the absence of a non-injured control group. Nevertheless, the cross-sectional design and the integration of clinical and genetic data allow the generation of meaningful hypotheses for future research. The principal strength of this study lies in its integrative approach, linking real-world clinical information with relevant genetic variation, thereby expanding current knowledge on biological susceptibility to knee ligament injuries in a context applicable to orthopedic practice.

## Conclusions

This study suggests that susceptibility to knee ligament injury is better explained by a cumulative polygenic profile than by any single collagen-gene polymorphism. While individual variants in COL1A1, COL1A2, and COL3A1 did not show strong independent effects, a higher combined genetic burden was associated with more complex injury patterns and a greater tendency toward non-contact mechanisms, consistent with the idea that subtle differences in connective tissue structure may influence how ligaments tolerate routine biomechanical loading. Overall, these findings support a multifactorial view of ligament injury risk and provide a rationale for future work exploring how genetic information might complement clinical and biomechanical assessment in prevention and patient counseling.

## Appendices

Clinical questionnaire

Project: Correlation Between Genetic Polymorphisms and Risk of Knee Ligament Injuries

Principal Investigator: Dr. Jorge Lara Arias

Thesis Author: Dr. Jürgen Frese Arroyo

Institution: Orthopedics and Traumatology Department, University Hospital “Dr. José Eleuterio González”, UANL

I. General Information

Participant name (initials or participant code): ______________________

Sex: ☐ Male ☐ Female

Age (years): ______

Occupation: __________________________________

Main sport/physical activity: ______________________________ ☐ Recreational ☐ Competitive

Lower-limb dominance: ☐ Right ☐ Left

Approximate date of injury (dd/mm/yy): _____________________

II. Personal and Sports History

Have you previously had an injury in the same knee? ☐ Yes ☐ No If “Yes”, specify the type of injury: ____________________________

Have you had injuries in other joints? ☐ Yes ☐ No Specify which: ___________________________________________

Physical activity level prior to the injury: ☐ Sedentary ☐ Moderate ☐ High ☐ Very high (regular athlete)

III. Mechanism and Injury Characteristics

How did the injury occur? ☐ Sports activity ☐ Fall ☐ Traffic accident ☐ Other: ____________

Severity of trauma: ☐ Mild ☐ Severe

Was the injury contact or non-contact? ☐ Contact ☐ Non-contact

On the day of injury, were you able to continue the activity? ☐ Yes ☐ No

Date of first medical consultation (dd/mm/yy): _____________

Clinical diagnosis provided: ______________________________

Diagnostic method: ☐ Clinical examination ☐ X-ray ☐ MRI ☐ Other: _______

Injured ligament(s) (per medical diagnosis): ☐ ACL ☐ PCL ☐ MCL ☐ LCL ☐ Multiple

IV. Treatment and Rehabilitation

Type of treatment received: ☐ Conservative ☐ Surgical

Did you receive rehabilitation therapy? ☐ Yes ☐ No If “Yes”: approximate duration (weeks): ________

Was the knee immobilized after the injury? ☐ Yes ☐ No If “Yes”: immobilization time (weeks): ________

Did you use crutches or other walking support? ☐ Yes ☐ No If “Yes”: for how many weeks: ________

Total time until medical discharge (weeks): ________

V. Outcome and Current Status

Were you able to return to sports or work after treatment? ☐ Yes ☐ No If “Yes”: approximate time to return (weeks): ________

If surgery was performed, did you have complications or require a second procedure? ☐ Yes ☐ No Specify: _________________________________________________

Current status of the injured knee (patient self-report):

Pain: ☐ None ☐ Mild ☐ Moderate ☐ Severe

Stability: ☐ Normal ☐ Mild instability ☐ Significant instability

Functional limitation: ☐ No ☐ Partial ☐ Significant

Have you had to modify your lifestyle or stop any physical activity/sport? ☐ Yes ☐ No Specify: ________________________________________________

VI. Family History

Do you have first-degree relatives (parents/siblings) who have had similar knee injuries? ☐ Yes ☐ No

Do any relatives practice high-impact sports or activities (soccer, basketball, athletics, dance)? ☐ Yes ☐ No Specify: ________________________________________________

VII. Investigator Notes

Investigator administering the questionnaire (signature):

____________________________
 

Date: ____ / ____ / ______
